# Carbapenems as water soluble organocatalysts

**DOI:** 10.12688/wellcomeopenres.14721.1

**Published:** 2018-08-31

**Authors:** Thomas L. Williams, Alexander R. Nödling, Yu-Hsuan Tsai, Louis Y. P. Luk

**Affiliations:** 1 Chemistry, Cardiff University, Cardiff, CF10 3AT, UK

**Keywords:** Organocatalysis, water-soluble iminium catalyst, carbapenem, Michael addition, artificial enzyme

## Abstract

**Background: **Identification of organocatalysts functioning in aqueous environments will provide methods for more sustainable chemical transformations and allow tandem reactions with biocatalysts, like enzymes. Here we examine three water-soluble carbapenem antibiotics (meropenem, doripenem, and ertapenem) as secondary amine organocatalysts in aqueous environments.

**Methods: **The Michael addition of nitromethane to cinnamaldehyde was used as the model reaction. The reactions were monitored by ^1^H NMR, and the enantioselectivity was determined by chiral HPLC.

**Results:** The effects of buffer components, pH, organic co-solvents and anchoring into a protein scaffold were investigated. Moderate yields of the Michael addition were obtained in buffer alone. The use of methanol as a co-solvent in a ratio of 1:1 increases the yield by 50%. Anchoring of the catalysts into a protein backbone reverses the enatioselectivity of the reaction.

**Conclusions:** Despite only moderate yields and enantioselectivities being obtained, this study lays the foundations for future development of efficient organocatalysis in aqueous environments.

## Introduction

**Figure 1.**
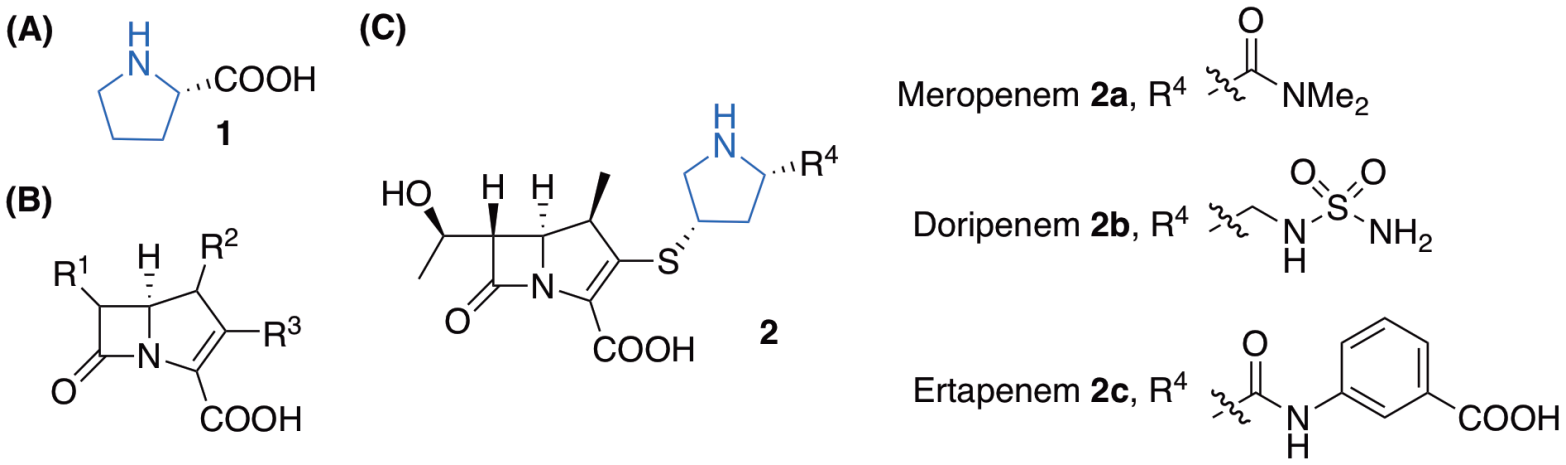
Structures of carbapenems. ( **A**) Structure of l-proline **1**. ( **B**) General structure of carbapenems **2**. ( **C**) Structure of meropenem **2a**, doripenem **2b** and ertapenem **2c**. Pyrrolidine moieties that contain a secondary amine are colored in blue.

The need for more sustainable catalysts in chemical transformations continues to attract significant interest ^1^. Iminium ion catalysis facilitated by secondary amines retrieved considerable attention in the early 2000’s due to their ability to activate enals for enantioselective nucleophilic addition ^2^. Now known as organocatalysts, these small molecules are reasonably cheap, non-toxic, sustainable, and stable (i.e. tolerant to moisture and air) ^3^. However, attempts to use organocatalysts in solvents like water as a homogenous system are yet to achieve great success ^4^. This is because most organocatalysts have bulky hydrophobic groups that are important for creating chiral environments, but significantly lower their water solubility ^5^. Not surprisingly, one of the most well-known water soluble organocatalysts is l-proline ( **1**, Figure 1A) that bears no bulky hydrophobic group. However, at neutral pH in water the proximity of the carboxylate to the amine of l-proline, making the amine more prone to be protonated and consequently inhibiting formation of the substrate iminium ion and leading to poor catalytic activity ^6^. In addition, the small chiral substituent in proline often hampers the enantioselectivity of the reactions.

**Figure 2.**
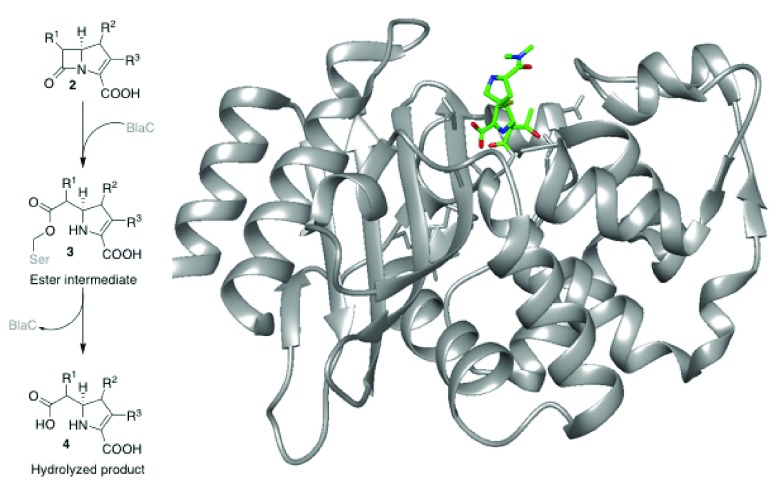
Metabolism of carbapenems 2 by penicillin binding proteins. ( **A**) Carbapenem metabolism. ( **B**) Crystal structure of intact meropenem **2a** in *Mycobacterium tuberculosis* β-lactamase BlaC, a penicillin binding protein, before metabolism; pdb: 3DWZ ^10^.

Besides proline, several carbapenems (e.g. meropenem **2a**, doripenem **2b**, and ertapenem **2c**; Figure 1) also have a secondary amine in the form of pyrrolidine. Carbapenems are β-lactam antibiotics that inhibit enzymes in bacterial cell wall biosynthesis ^7^. Just like proline, carbapenems are completely water soluble. However, the lack of an adjacent carboxylate group in carbapenems implies that these molecules should be able to form an iminium ion readily in aqueous environments for efficient catalysis. On the other hand, substituents around the pyrrolidine ring of carbapenems are larger than that of proline and likely to induce enantioselectivity for the catalysis ^8^. In addition, an enhanced chiral environment can be created by anchoring carbapenems into a protein environment. For example, a covalent adduct **3** ( Figure 2) is formed during the metabolism of carbapenems by penicillin binding proteins, and this intermediate can be trapped by mutating the key glutamic acid residue to an alanine residue to prevent the hydrolysis of the ester intermediate ^9^.

Here we explored the uses of three carbapenem antibiotics, meropenem **2a**, doripenem **2b** and ertapenem **2c**, as organocatalysts in aqueous systems. The effects of buffer components, pH, and organic co-solvents on reaction yield were investigated. We also tested whether anchoring a carbapenem in a protein scaffold increases the enantioselectivity of the reaction.

## Methods

### General procedure for the Michael addition of nitromethane to cinnamaldehyde

For the buffer screen, a concentration of 10 mM at pH 7.0 was used for each of the following: (4-(2-hydroxyethyl)-1-piperazineethanesulfonic acid (HEPES), potassium phosphate (KPi), sodium phosphate (NaPi), or phosphate-buffered saline (PBS, i.e. sodium phosphate and sodium chloride). For doripenem reactions, to a solution of doripenem (Glentham Life Sciences #GA9212) (1.00 mg, 2.60 µmol, 1 eq) in the indicated buffer (495 µl) was added cinnamaldehyde (1.57 mg, 11.9 µmol, 5 eq) in MeOH (5 µl) and nitromethane (1.27 µl, 23.8 µmol, 10 eq). For meropenem reactions, to a solution of meropenem trihydrate (Ark Pharm Inc, #AK161987) (1.14 mg, 2.38 µmol, 1 eq) in the indicated buffer (495 µl) was added cinnamaldehyde (1.72 mg, 13.0 µmol, 5 eq) in MeOH (5 µl) and nitromethane (1.39 µl, 26.0 µmol, 10 eq). For ertapenem reactions, to a solution of ertapenem sodium salt (Glentham Life Sciences, #GA8176) (1.04 mg, 2.10 µmol, 1 eq) in the indicated buffer (495 µl) was added cinnamaldehyde (1.39 mg, 10.5 µmol, 5 eq) in MeOH (5 µl) and nitromethane (1.12 µl, 21.0 µmol, 10 eq). The reactions were then placed in a thermoshaker at 25°C and shaken at 800 rpm for 24 hours.

For reactions carried out at pH 7.5 and pH 8.0, the buffers were adjusted with 1 M NaOH, and the pH measured using a pH meter. For the co-solvent screen the reactions were performed by first adding 245 µl of the indicated buffer followed by 250 µl of the indicated solvent.

For reactions in buffer alone, the reaction mixtures were extracted with 700 µl of CDCl _3_ and analyzed by ^1^H NMR spectroscopy. For those reactions with methanol or acetonitrile as the co-solvent, the solvents were evaporated under reduced pressure, and the crude mixture was extracted with 700 µl of CDCl _3_. Reactions containing benzene or chloroform as the co-solvent were performed using the corresponding deuterated solvents and subjected directly to NMR analysis. Product yields were estimated from integration of signals arising from cinnamaldehyde **6**, product **7**, and side product **8**. ^1^H NMR spectra were recorded in CDCl _3_ or C _6_D _6_ on a Bruker Ascend 500 MHz or a Bruker Fourier 300 MHz instrument. Chemical shifts are reported in parts per million (ppm) and are referenced to the residual solvent resonance as the internal standard (CHCl _3_: δ = 7.26 ppm and C _6_H _6_: δ = 7.15 ppm). Spectra were analysed using Bruker TopSpin version 3.5 ^11^.

The following conditions were used to carry out the modified (diluted) BlaC-carbapenem reactions. To a microcentrifuge tube was added 250 µl of the enzyme solution (10 mg, 324 nmol, 0.2 eq) and to this was added 245 µl of reaction buffer (50 mM NaP _i_, 100 mM NaCl, pH 7.0). To the enzyme was added 5 µl of a stock solution of cinnamaldehyde in methanol, (213 µg, 1.6 µmol, 1 eq) followed by 0.17 µl (195.2 µg, 3.2 µmol, 2 eq) of neat nitromethane. The reactions were shaken at 50 rpm, 25°C for 24 h. 700 µl of CDCl _3_ was added to the reactions when finished and the samples spun down in a micro centrifuge. The organic fraction was removed and subjected to ^1^H NMR analysis. The control reactions carried out in tandem with meropenem **2a** and doripenem **2b** were performed as follows. For doripenem reactions, to a solution of doripenem (136 µg, 324 nmol, 1.0 eq) in PBS buffer (495 µl) was added cinnamaldehyde (213 µg, 1.6 µmol, 5 eq) in MeOH (5 µl) and nitromethane (3.2 µmol, 2 eq). For meropenem reactions, to a solution of meropenem trihydrate (124 µg, 324 nmol, 1 eq) in PBS buffer (495 µl) was added cinnamaldehyde (213 µg, 1.6 µmol, 5 eq) in MeOH (5 µl) and nitromethane 0.17 µl (3.2 µmol, 2 eq).

### Determination of enantioselectivity

The stereoselectivity of products **7** were determined by chiral high-performance liquid chromatography (HPLC) analysis. Enantioenriched samples of ( *S*)- and ( *R*)-nitro products for peak assignment in chiral HPLC measurements were obtained using l **-** and d-Jørgensen-Hayashi catalysts according to the literature procedure ^5^. A racemic sample of the nitro product was obtained using piperidine as the catalyst ^12^. The aldehyde was reduced to the alcohol for determining the ratio of two enantiomers ( Scheme S1).

The reactions were scaled up 10 times with respect to the above screening conditions for the reactions with meropenem and doripenem in pH 7.0 PBS buffer and with 50% methanol and performed in 10-ml round bottom flasks with continuous stirring at room temperature for 24 h. The protein reactions were scaled up 10 times with respect to the reactions used for screening.

The reaction mixture was then extracted with DCM (10 ml × 3), and the organic fractions were combined, dried over anhydrous Na _2_SO _4_, filtered and concentrated under reduced pressure to ca. 1 ml before purification by preparative TLC (EtOAc:hexane = 25:75). The silica gel was scraped from the plate and stirred in 1% MeOH:DCM (10 ml). The suspension was filtered and evaporated under reduced pressure.

The resulting aldehyde was dissolved in methanol (5 ml), and to this was added ca. 5 equivalents of NaBH _4_. The reaction was stirred overnight at room temperature. The reaction mixture was then neutralized with 1 M HCl _(aq)_, and then extracted with DCM (10 ml x 3). The organic fractions were combined, dried over Na _2_SO _4_, filtered and concentrated under reduced pressure. The resulting alcohol was purified by preparative TLC (EtOAc:hexane = 35:65). The silica was scraped from the plate and stirred in 1% MeOH:DCM (10 ml). The solution was filtered and evaporated under vacuum.

The purified alcohol was dissolved in 1 ml of 20% 2-propanol in hexane (HPLC grade). 20 µl of each sample was injected onto the HPLC.

The reactions catalyzed by meropenem **2a** and doripenem **2b** were scaled up ten times from the above conditions and the reaction mixtures were extracted with dichloromethane, dried over Na _2_SO _4_, filtered, and concentrated. The product **7** was purified by preparative TLC (25% EtOAc in hexane). The product was dissolved in 5 ml of methanol, and approx. 5 equivalents of NaBH _4_ were added. After overnight stirring at room temperature, the reaction mixture was neutralized with 1 M HCl _(aq)_, extracted with dichloromethane (3 × 10 ml), dried over Na _2_SO _4_, filtered, and concentrated. The resulting alcohol **9** was purified by preparative TLC (35% EtOAc in hexane) before analysis by chiral HPLC using a Phenomonex Cellulose-Lux1 analytical chiral column held at 20°C (isocratic elution 0.5 ml/min with 25% 2-propanol in *n*-hexane; detection by absorbance at 210 nm).

To determine the enantioselectivity of the BlaC-carbapenem complexes, the reactions were scaled up 10 times with regards to the small-scale screening reactions. Enantioselectivity was then determined using the above method.

### Cloning, expression and purification of BlaC(E166A)

The gene encoding for the wild-type *Mycobacterium tuberculosis* β-lactamase (BlaC) without the 40-amino acid leader sequence was purchased as a double-stranded fragment (GeneArt, Invitrogen; see Supporting Information for the exact DNA sequence). This was cloned into a NdeI and BamHI digested pET28a vector by Gibson assembly to yield the wild type gene with an N-terminal 6 his-tag originating from the vector. Briefly, 25 ng of the linear gene was added to 100 µg of digested plasmid and to this was added 0.5 µl of sterile H _2_O followed by 2.5 µl of Gibson assembly master mix (NEB). The mixture was then incubated at 50°C for 1 hour. The products were transformed into chemically competent MDS42 *E. coli* cells and grown overnight. Colonies were selected and cultured overnight after which the plasmid was purified by the miniprep method (Qiagen). The construct was confirmed by DNA sequencing.

The E166A mutation was introduced by site-directed mutagenesis PCR using PrimeStar HS DNA polymerase (Clonetech) with primers CTGGATGCAGAAGCACCGGAACTGAATC (forward) and GATTCAGTTCCGGTGCTTCTGCATCCAG (reverse). PCR was performed over 33 cycles. The initial temperature was 95°C and held for 3 minutes. Each cycle comprised of 10 seconds for denaturation at 98°C, followed by annealing for 5 seconds at 60°C and finally an extension of 6 minutes and 30 seconds at 72°C. The final extension was 10 minutes. The mutant construct was confirmed by DNA sequencing (Eurofins, Genomics) using the T7 promoter primer (TAATACGACTCACTATAGG).

Plasmid pET28a BlaC(E166A) was then transformed into chemically competent BL21(DE3) cells and grown on LB agar plates supplemented with kanamycin (37.5 µg/ml). One colony from the plate was picked to inoculate a 10 ml LB starter culture containing kanamycin (37.5 µg/ml). After overnight, the starter culture was diluted into 1 L of fresh LB media containing kanamycin (37.5 µg/ml). The cells were grown at 37°C until they reached an OD _600_ of 0.8, and IPTG was added to reach a final concentration of 0.5 mM. The cells were then incubated overnight at 20°C. The cultures were harvested and the dry pellet was stored at –20°C.

**Figure 3.**
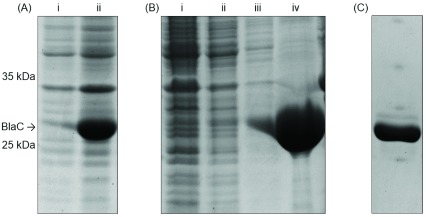
SDS-PAGE analysis for the expression and purification of BlaC. ( **A**) Expression of BlaC: i, before induction; ii, after induction. ( **B**) Nickel affinity chromatography of BlaC-meropenem complex **3a**: i, flow through; ii, wash 1; iii, wash 2, iv, elution. ( **C**) Elution of BlaC-meropenem complex **3a** from size exclusion chromatography.

To purify the enzyme, the cells were lysed by sonication in sodium phosphate buffer (35 ml per litre pellet, 50 mM NaPi, 100 mM NaCl, 10 mM imidazole, pH 8.0) with either 5 mg meropenem or doripenem added to the re-suspended cells. The solids were separated by centrifugation at 20,000 rpm for 30 minutes. The supernatant was loaded on to a Ni-NTA column equilibrated with the above buffer. The column was washed twice with wash buffer (25 ml, 50 mM NaPi, 100 mM NaCl, 25 mM imidazole, pH 8.0) and then the protein was eluted in phosphate buffer (10 ml, 50 mM NaPi, 100 mM NaCl, 300 mM imidazole, pH 8.0). 1 mg of doripenem or meropenem was added to the elution fraction and incubated for 30 minutes at room temperature. Fast protein liquid chromatography (FPLC) was performed on an ÄKTA purifier (GE Healthcare) system at room temperature using a ProteoSEC size exclusion column (Generon, SEC-3-70-100 ml, 26 mm ID, 60 cm length, 3–70 kDa HR resin). Protein elution was monitored by UV absorbance at 280 nm and the elution buffer was 50 mM sodium phosphate buffer 100 mM NaCl pH 7.0. Fractions containing BlaC-carbapenem complexes **3** were combined and concentrated using a 10 kDa cutoff centrifugal concentrator. Expression and purification were confirmed by sodium dodecyl sulfate–polyacrylamide gel electrophoresis (SDS-PAGE) ( Figure 3). SDS-PAGE was performed using self-casted 12% acrylamide gels and run for 50 minutes at 200 V. SDS-PAGE gels were stained using Coomassie Brilliant Blue. Unstained protein molecular weight marker (Thermo Scientific) was run alongside the samples.

 Protein-carbapenem complexes were confirmed by Liquid chromatography mass spectrometry (LC-MS). LC-MS was performed on a Waters Synapt G2-Si quadrupole time of flight mass spectrometer coupled to a Waters Acquity H-Class UPLC system. The column was an Acquity UPLC protein BEH C4 (300 Å 1.7 µm × 2.1 mm × 100 mm) operated in reverse phase and held at 60°C. The gradient employed was 95% A to 35% A over 50 minutes, where A is H _2_O with 0.1% HCO _2_H and B is acetonitrile (ACN) with 0.1% HCO _2_H. Data was collected in positive ionization mode and analysed using the Waters MassLynx software version 4.1. Deconvolution of protein charged states was obtained using the maximum entropy 1 processing software.

## Results and discussion

**Scheme 1.**
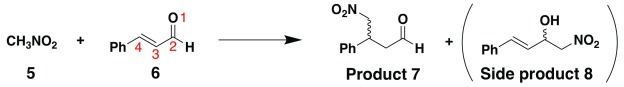
Michael addition of nitromethane **5** to cinnamaldehyde **6** as the model reaction resulting in the 1,4-addition product **7** and sometimes the 1,2-addition side product **8**.

Here we chose the Michael addition of nitromethane **5** to cinnamaldehyde **6** ( Scheme 1) as the model reaction to test whether meropenem **2a**, doripenem **2b**, and ertapenem **2c** can function as catalysts. We first investigated different buffer conditions ( Table 1). In the presence of 20% catalyst, all four buffers produced similar yields with each catalyst, but significantly higher yields were observed for meropenem **2a** and doripenem **2b** in comparison to ertapenem **2c**. It is noteworthy that no product was formed in non-buffered conditions (i.e. pure water), suggesting the need for a controlled pH throughout the catalytic cycle ^13^. On the other hand, the presence of NaCl in PBS buffer had minimal impact on product yield. Since PBS is a common buffer used to mimic biological environments, this buffer was chosen for further investigations.

**Table 1.** Percentage yields of 7 from the model reaction with different buffers at pH 7.0. Yields were estimated using ^1^H NMR.

Catalyst	H _2_O	HEPES	KP _i_	NaP _i_	PBS
Meropenem **2a**	0	33	38	33	35
Doripenem **2b**	0	38	29	39	31
Ertapenem **2c**	1	8	9	8	13

### Modification of pH

We then shifted our attention to modify the reaction pH ( Table 2). PBS adjusted to the relevant pH was employed. Although the conversion of the starting material **6** seems to increase at the elevated pH, the yield of the product did not. We found that this was due to formation of the side product **8** by 1,2-addition of nitromethane to cinnamaldehyde **6**. As side product formation was least prominent at pH 7.0, we determined that this is the preferred pH for the reaction.

**Table 2.** Percentage yields of product 7 and side product 8 in 10 mM PBS at indicated pH. Yields were estimated using ^1^H NMR.

Catalyst	pH 7.0		pH 7.5		pH 8.0	
	7	8	7	8	7	8
Meropenem **2a**	35	1	25	75	23	25
Doripenem **2b**	31	1	34	26	29	28
Ertapenem **2c**	13	0	8	4	5	3

### Optimization of yield

To optimize the reaction yield, we decided to explore the effects of different organic solvents as a co-solvent ( Table 3). Four solvents were chosen and tested in a 1:1 ratio to the PBS buffer (10 mM, pH 7.0). These solvents are either miscible (methanol and acetonitrile) or non-miscible (benzene and chloroform) with the buffer. Addition of methanol was found to significantly increase the product yield, whereas acetonitrile had negligible effects, and the use of the non-miscible solvents totally abolished the product formation.

**Table 3.** Percentage yields of 7 from the model reaction in the presence of different co-solvents. Yields were estimated using ^1^H NMR.

Catalyst	Miscible co-solvent		Non-miscible co-solvent	
	Methanol	Acetonitrile	Benzene	Chloroform
Meropenem **2a**	71	19	0	0
Doripenem **2b**	74	27	1	2
Ertapenem **2c**	22	5	0	6

As reasonable yields could be obtained using either meropenem **2a** or doripenem **2b** as the catalyst, chiral HPLC was employed to identify the enantioselectivity under these conditions. Reactions were performed on a larger scale to facilitate product purification and isolation by preparative TLC. The aldehyde functionality in the product **7** was reduced by treatment with NaBH _4_ to afford the corresponding alcohol **9** before analysis by chiral HPLC (see Supporting Information) ^5^. In general, no significant difference in the enantioselectivity was observed for either meropenem **2a** or doripenem **2b** ( Table 4), and the addition of methanol also had little effect. In fact, enantiomeric excess is low in all cases, with the best result of *R*: *S* = 41:59 observed with meropenem **3a** in the mixture of PBS and methanol.

**Table 4.** Enantiomeric ratio ( *R*: *S*) of the product 7. Ratio calculated from integration of chiral high-performance liquid chromatography peaks.

Catalyst	PBS only	1:1 PBS:MeOH
Meropenem **2a**	43:57	41:59
Doripenem **2b**	43:57	47:53

### Improving enantioselectivity

**Figure 4.**
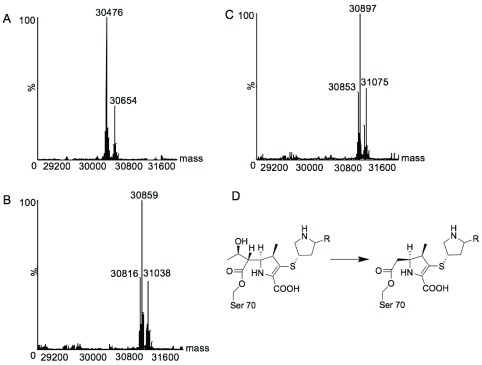
Deconvoluted ESI-MS of purified BlaC enzymes. ( **A**) BlaC reference mass = 30476 Da (30654 Da = BlaC + F-Met) ( **B**) BlaC + meropenem = 30859 Da (31038 Da = BlaC + meropenem + F-Met, 30816 Da = retro aldol product of meropenem bound to BlaC) ( **C**) BlaC + doripenem = 30897 Da (31075 Da = BlaC + doripenem + F-Met, 30853 Da = retro aldol product of doripenem bound to BlaC) ( **D**) Retro aldol product of the carbapenems.

To improve the enantioselectivity, we anchored meropenem **2a** or doripenem **2b** into the *M. tuberculosis* β-lactamase BlaC. This enzyme is known to metabolize carbapenems ( Figure 2A). However, mutation of Glu166 to Ala can prevent hydrolysis of the ester intermediate **3** ^14^, enabling stable anchoring of the antibiotics to BlaC. Recombinant BlaC(E166A) containing a N-terminal His-tag was produced in *Escherichia coli* BL21(DE3). Cells were lysed in the presence of meropenem **2a** or doripenem **2b**, and the protein complex was purified by nickel affinity and size exclusion chromatography ( Figure 3). Formation of the protein complexes **3a** and **3b** with covalently bound ester intermediate were confirmed by mass spectrometry ( Figure 4).

The Michael reaction was then performed with the BlaC-carbapenem complexes **3a** and **3b**. Attempts to perform reactions with BlaC-carbapenem **3a** or **3b** using the experimental conditions in Table 4 were not successful as the enzyme complexes precipitated in buffers containing > 10% (v/v) methanol or if the concentration of the BlaC-carbapenem complex **3** exceeded 500 µM in PBS. Thus, we were not able to test the conditions giving the highest yields (i.e. 1:1 = PBS: MeOH), and the reactions were carried out at diluted concentration in PBS ( Table 5). Under the new conditions, we were able to maintain the ratio of BlaC-carbapenem complexes (20 mol% **3**) to cinnamaldehyde **6** and negligible precipitation of protein complexes was observed during the time course of the reaction. Under the new (diluted) condition, as expected, lower yields were obtained with either meropenem **2a** or doripenem **2b** compared to the previous conditions ( Table 1, Table 5). While no product was obtained in the presence of BlaC only, some products were obtained in the presence of BlaC-meropenem **3a** or BlaC-doripenem **3b**complexes, although there was no significant change in product yield compared to the carbapenems **2** alone ( Table 5). Notably, inversion of enantioselectivity was observed when the catalyst was anchored in the protein scaffold, although the enantioselectivity is still low. Raw data for all experiments conducted in this study, including the supporting information, are available on figshare ^15^.

**Table 5.** Percentage yields and enantiomeric ratio of 7 in the presence of BlaC-carbapenem complexes 3. Yields were estimated using ^1^H NMR. Ratio calculated from integration of chiral high-performance liquid chromatography peaks.

Catalyst	Yield (%)	*R*: *S*
Meropenem **2a**	22	43:57
BlaC-meropenem **3a**	20	55:45
Doripenem **2b**	23	46:54
BlaC-doripenem **3b**	27	58:42

## Conclusions

Here we investigated the ability of carbapenem antibiotics **2a-c** to catalyze the Michael addition of nitromethane to cinnamaldehyde **6** in aqueous environments. While ertapenem **2c** is not effective, catalysis by meropenem **2a** or doripenem **2b** provides the product **7** with moderate yields and enantioselectivities.

The results suggest the carbapenems **2a-c** can form iminium ions *in situ*, facilitating the nucleophilic addition of nitromethane for Michael addition in water. The charged iminium intermediate may be stabilized by polar protic solvents (e.g. water, methanol). This is consistent with the observation that higher yields were obtained when using methanol as a co-solvent. On the other hand, polar aprotic solvents (e.g. acetonitrile) had little effect. Addition of non-miscible solvents (e.g. benzene, chloroform) into the buffer system was not beneficial for catalysis, most likely due to the preferential partition of the starting materials into the organic phase, whereas the catalyst remains in the aqueous phase, hindering catalysis.

It does not seem that the substitution around the active nitrogen is enough to induce high enantioselectivity onto the product at 25°C. Attack from either the *re* or *si* face of the iminium ion seems to be equally possible for meropenem **2a** and doripenem **2b**.

The crystal structure of the BlaC-carbapenem complex ( Figure 2) suggests that access to the amine by the substrates is still possible when meropenem **2a** is bound to BlaC. Indeed, anchoring the carbapenems **2a** and **2b** into the protein produced similar yields to the catalysts alone. However, an inversion of stereoselectivity was observed for the BlaC-carbapenem complexes **3**, suggesting that the protein environment surrounding the catalyst determines the enantioselectivity. The low enantioselectivity may be due to the sub-optimal local environment provided by the wild-type protein, and engineering of this pocket by mutagenesis may increase both reaction yields and enantioselectivities, as demonstrated previously in artificial metalloenzymes ^16^.

In this study, we have shown that water-soluble carbapenem antibiotics **2a**- **c** can be repurposed to catalyze Michael addition. Unlike most organocatalysts used in aqueous systems ^17^, these catalysts do not require emulsion or biphasic systems. Separation of the catalysts from the product can be achieved easily by extraction without the need for chromatography. Identification of organocatalysts functioning in aqueous environments will provide more sustainable approaches for chemical transformations and allow tandem reactions with biocatalysts, such as enzymes. However, there exists room to improve the system reported here, as only moderate yields and enantioselectivities were obtained. Nonetheless, this study lays the foundations toward developing efficient iminium catalysis in water and provides new strategies of anchoring small molecule catalysts into chiral protein environments.

## Data availability

The H NMR spectra, chiral HPLC chromatograms and full protein LC-MS chromatograms, and mass spectra datasets generated during and/or analyzed during the current study are available in the figshare repository: https://doi.org/10.6084/m9.figshare.6973880 ^15^.

## Supplementary material

**Supporting information. Further results supporting the conclusions of this article.** Included are spectra for Michael addition reactions, chiral HPLC chromatograms, relevant DNA and amino acid sequences and LC-MS analysis of BlaC/BlaC-carbapenem complexes.
