# Supplementary material for: Carbapenems as water soluble organocatalysts
Source: Wellcome Open Res. 2018 Aug 31;3:107. [Version 1] doi: 10.12688/wellcomeopenres.14721.1 (PMC6198258; doi:10.12688/wellcomeopenres.14721.1)
Supplement: Supplementary file 1 [file wellcomeopenres-3-16037-s0000.tgz › 78544d6a-7562-4bb2-aaf8-e1512a854c80_Wellcome_SI.docx]

Carbapenems as water soluble organocatalysts

Thomas L. Williams, Alexander R. Nödling, Yu-Hsuan Tsai and Louis Y. P. Luk^*^

Address: School of Chemistry, Cardiff University, Main Building, Park Place, Cardiff CF10 3AT, United Kingdom.

Email: LukLY@cardiff.ac.uk

*Corresponding author

**Supplementary information**

# **Nitro-Michael addition reactions**

All screening reactions in Tables 1-3 were carried out in triplicate.

## **1.1 Carbapenem screening reactions**

Cinnamaldehyde (Red) and Nitro addition product (Blue) in CDCl_3_ reference spectra

Doripenem in H_2_O – Run 1

Doripenem in H_2_O – Run 2

Doripenem in H_2_O – Run 3

Doripenem in HEPES (pH 7.0) buffer – Run 1

Doripenem in HEPES (pH 7.0) buffer – Run 2

Doripenem in HEPES (pH 7.0) buffer – Run 3

Doripenem in KP_i_ (pH 7.0) buffer – Run 1

Doripenem in KP_i_ (pH 7.0) buffer – Run 2

Doripenem in KP_i_ (pH 7.0) buffer – Run 3


Doripenem in NaP_i_ (pH 7.0) buffer – Run 1

Doripenem in NaP_i_ (pH 7.0) buffer – Run 2

Doripenemin NaP_i_ (pH 7.0) buffer – Run 3

Doripenem in PBS (pH 7.0) buffer – Run 1

Doripenem in PBS (pH 7.0) buffer – Run 2

Doripenem in PBS (pH 7.0) buffer – Run 3

Doripenem in PBS (pH 7.5) buffer – Run 1

Doripenem in PBS (pH 7.5) buffer – Run 2

Doripenem in PBS (pH 7.5) buffer – Run 3

Doripenem in PBS (pH 8.0) buffer – Run 1

Doripenem in PBS (pH 8.0) buffer – Run 2

Doripenem in PBS (pH 8.0) buffer – Run 3

Doripenem in 50:50 PBS (pH 7.0) buffer:MeOH – Run 1

Doripenem in 50:50 PBS (pH 7.0) buffer:MeOH – Run 2

Doripenem in 50:50 PBS (pH 7.0) buffer:MeOH – Run 3

Doripenem in 50:50 PBS (pH 7.0) buffer:ACN – Run 1

Doripenem in 50:50 PBS (pH 7.0) buffer:ACN – Run 2

Doripenem in 50:50 PBS (pH 7.0) buffer:ACN – Run 3

Doripenem in 50:50 PBS (pH 7.0) buffer:CDCl_3_ – Run 1

Doripenem in 50:50 PBS (pH 7.0) buffer:CDCl_3_ – Run 2

Doripenem in 50:50 PBS (pH 7.0) buffer:CDCl_3_ – Run 3

Doripenem in 50:50 PBS (pH 7.0) buffer:C_6_D_6_ – Run 1

Doripenem in 50:50 PBS (pH 7.0) buffer:C_6_D_6_ – Run 2

Doripenem in 50:50 PBS (pH 7.0) buffer:C_6_D_6_ – Run 3

Meropenem in H_2_O – Run 1

Meropenem in H_2_O – Run 2

Meropenem in H_2_O – Run 3

Meropenem in HEPES (pH 7.0) buffer – Run 1

Meropenem in HEPES (pH 7.0) buffer – Run 2

Meropenem in HEPES (pH 7.0) buffer – Run 3

Meropenem in KP_i_ (pH 7.0) buffer – Run 1

Meropenem in KP_i_ (pH 7.0) buffer – Run 2

Meropenem in KP_i_ (pH 7.0) buffer – Run 3

Meropenem in NaP_i_ (pH 7.0) buffer – Run 1

Meropenem in NaP_i_ (pH 7.0) buffer – Run 2

Meropenem in NaP_i_ (pH 7.0) buffer – Run 3

Meropenem in PBS (pH 7.0) buffer – Run 1

Meropenem in PBS (pH 7.0) buffer – Run 2

Meropenem in PBS (pH 7.0) buffer – Run 3

Meropenem in PBS (pH 7.5) buffer – Run 1

Meropenem in PBS (pH 7.5) buffer – Run 2

Meropenem in PBS (pH 7.5) buffer – Run 3

Meropenem in PBS (pH 8.0) buffer – Run 1

Meropenem in PBS (pH 8.0) buffer – Run 2

Meropenem in PBS (pH 8.0) buffer – Run 3

Meropenem in 50:50 PBS (pH 7.0) buffer:MeOH – Run 1

Meropenem in 50:50 PBS (pH 7.0) buffer:MeOH – Run 2

Meropenem in 50:50 PBS (pH 7.0) buffer:MeOH – Run 3

Meropenem in 50:50 PBS (pH 7.0) buffer:ACN – Run 1

Meropenem in 50:50 PBS (pH 7.0) buffer:ACN – Run 2

Meropenem in 50:50 PBS (pH 7.0) buffer:ACN – Run 3

Meropenem in 50:50 PBS (pH 7.0) buffer:CDCl_3_ – Run 1

Meropenem in 50:50 PBS (pH 7.0) buffer:CDCl_3_ – Run 2

Meropenem in 50:50 PBS (pH 7.0) buffer:CDCl_3_ – Run 3

Meropenem in 50:50 PBS (pH 7.0) buffer:C_6_D_6_ – Run 1

Meropenem in 50:50 PBS (pH 7.0) buffer:C_6_D_6_ – Run 2

Meropenem in 50:50 PBS (pH 7.0) buffer:C_6_D_6_ – Run 3

Ertapenem in H_2_O – Run 1

Ertapenem in H_2_O – Run 2

Ertapenem in H_2_O – Run 3

Ertapenem in HEPES (pH 7.0) buffer – Run 1

Ertapenem in HEPES (pH 7.0) – Run 2

Ertapenem in HEPES (pH7.0) buffer – Run 3

Ertapenem in KP_i_ (pH 7.0) buffer – Run 1

Ertapenem in KP_i_ (pH 7.0) buffer – Run 2

Ertapenem in KP_i_ (pH 7.0) buffer – Run 3

Ertapenem in NaP_i_ (pH 7.0) buffer – Run 1

Ertapenem in NaP_i_ (pH 7.0) buffer – Run 2

Ertapenem in NaP_i_ (pH 7.0) buffer – Run 3

Ertapenem in PBS (pH 7.0) buffer – Run 1

Ertapenem in PBS (pH 7.0) buffer – Run 2

Ertapenem in PBS (pH 7.0) buffer – Run 3

Ertapenem in PBS (pH 7.5) buffer – Run 1

Ertapenem in PBS (pH 7.5) buffer – Run 2

Ertapenem in PBS (pH 7.5) buffer – Run 3

Ertapenem in PBS (pH 8.0) buffer – Run 1

Ertapenem in PBS (pH 8.0) buffer – Run 2

Ertapenem in PBS (pH 8.0) buffer – Run 3

Ertapenem in 50:50 PBS (pH 7.0) buffer:MeOH – Run 1

Ertapenem in 50:50 PBS (pH 7.0) buffer:MeOH – Run 2

Ertapenem in 50:50 PBS (pH 7.0) buffer:MeOH – Run 3

Ertapenem in 50:50 PBS (pH 7.0) buffer:ACN – Run 1

Ertapenem in 50:50 PBS (pH 7.0) buffer:ACN – Run 2

Ertapenem in 50:50 PBS (pH 7.0) buffer:ACN – Run 3

Ertapenem in 50:50 PBS (pH 7.0) buffer:CDCl_3_ – Run 1

Ertapenem in 50:50 PBS (pH 7.0) buffer:CDCl_3_ – Run 2

Ertapenem in 50:50 PBS (pH 7.0) buffer:CDCl_3_ – Run 3

Ertapenem in 50:50 PBS (pH 7.0) buffer:C_6_D_6_ – Run 1

Ertapenem in 50:50 PBS (pH 7.0) buffer:C_6_D_6_ – Run 2

Ertapenem in 50:50 PBS (pH 7.0) buffer:C_6_D_6_ – Run 3

## BlaC-carbapenem screening reactions

BlaC-meropenem in PBS (pH 7.0) buffer

BlaC-doripenem in PBS (pH 7.0) buffer

Doripenem control in PBS (pH 7.0) buffer

Meropenem control in PBS (pH 7.0) buffer

# Chiral HPLC chromatograms

Racemic standard


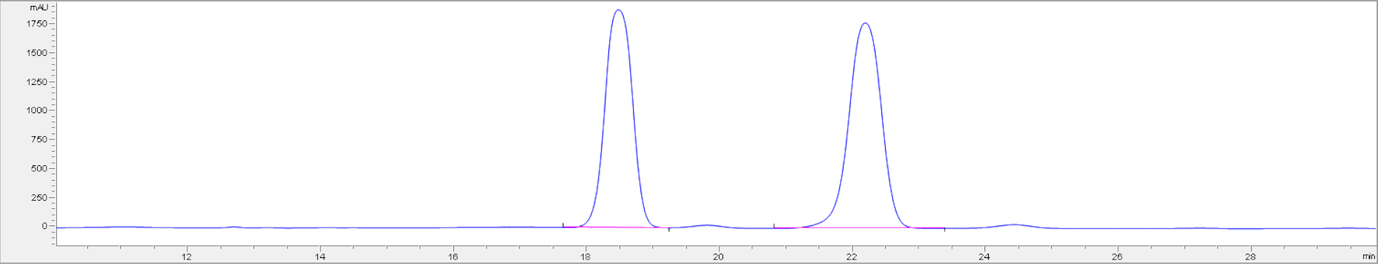


(*S*)-

(*R*)-

| Peak | Rt (Min) | Area (mAU*s) | Area (%) |
| --- | --- | --- | --- |
| 1 | 18.5 | 53631.9 | 47.2 |
| 2 | 22.2 | 60200.8 | 52.8 |

Meropenem in PBS (pH 7.0) buffer


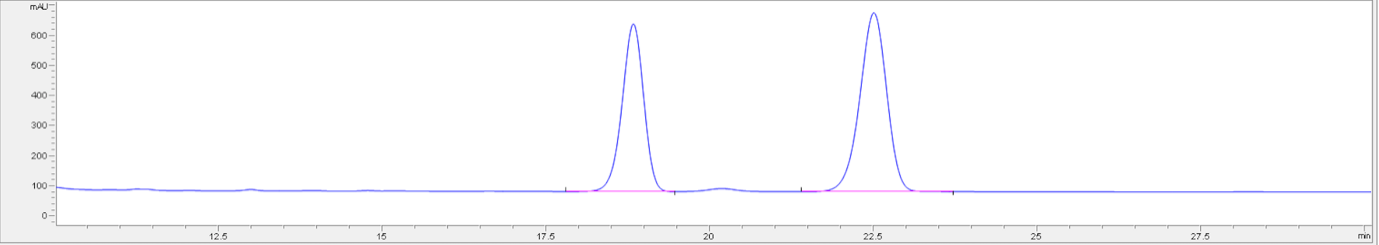


| Peak | Rt (Min) | Area (mAU*s) | Area (%) |
| --- | --- | --- | --- |
| 1 | 18.8 | 12791.9 | 42.9 |
| 2 | 22.5 | 17005.7 | 57.1 |

Doripenem in PBS (pH 7.0) buffer


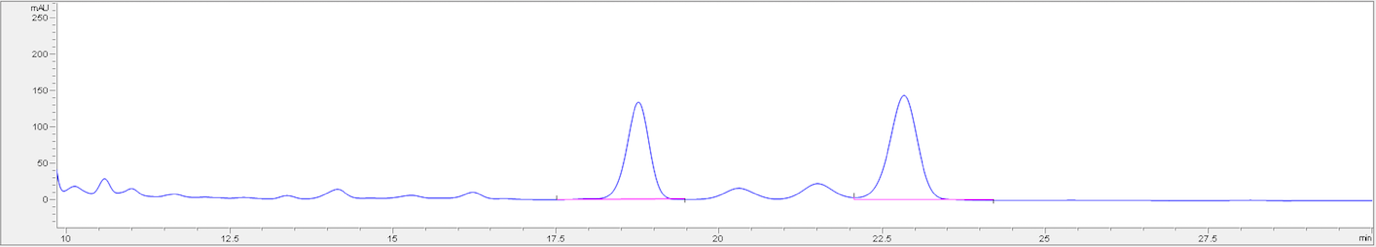


| Peak | Rt (Min) | Area (mAU*s) | Area (%) |
| --- | --- | --- | --- |
| 1 | 18.8 | 3277.4 | 42.7 |
| 2 | 22.8 | 4339.2 | 57.3 |

Meropenem in 50:50 PBS (pH 7.0) buffer:MeOH


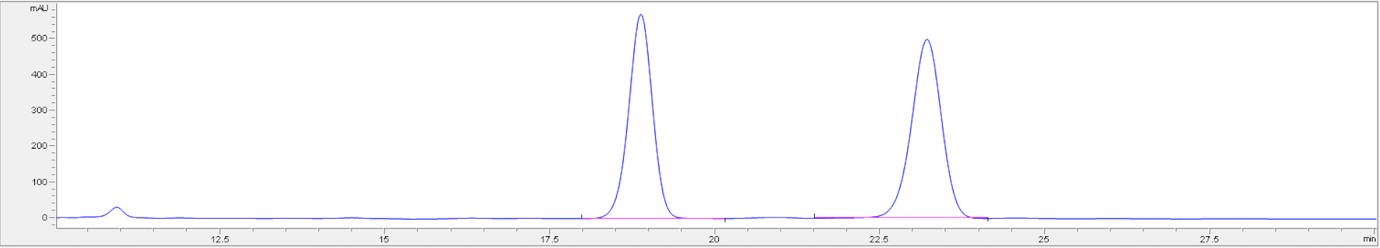


| Peak | Rt (Min) | Area (mAU*s) | Area (%) |
| --- | --- | --- | --- |
| 1 | 18.9 | 1855.8 | 41.3 |
| 2 | 23.1 | 2638.8 | 58.7 |

Doripenem – 50:50 PBS (pH 7.0) buffer:MeOH


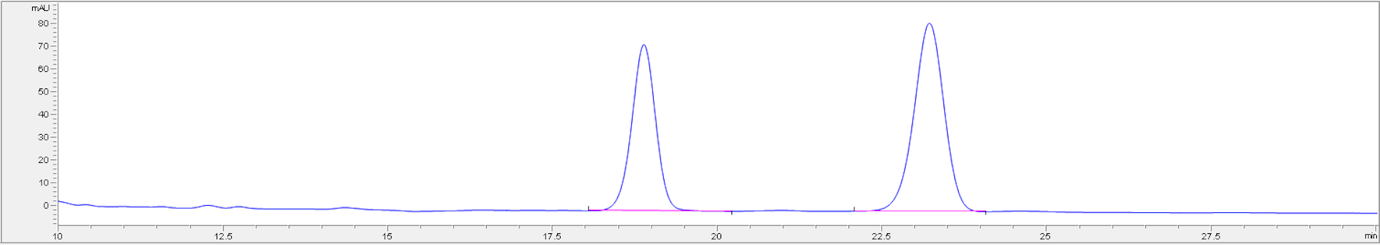


| Peak | Rt (Min) | Area (mAU*s) | Area (%) |
| --- | --- | --- | --- |
| 1 | 18.9 | 14448.3 | 47.4 |
| 2 | 23.1 | 16012.0 | 52.6 |

Meropenem – BlaC complex in PBS (pH 7.0) buffer


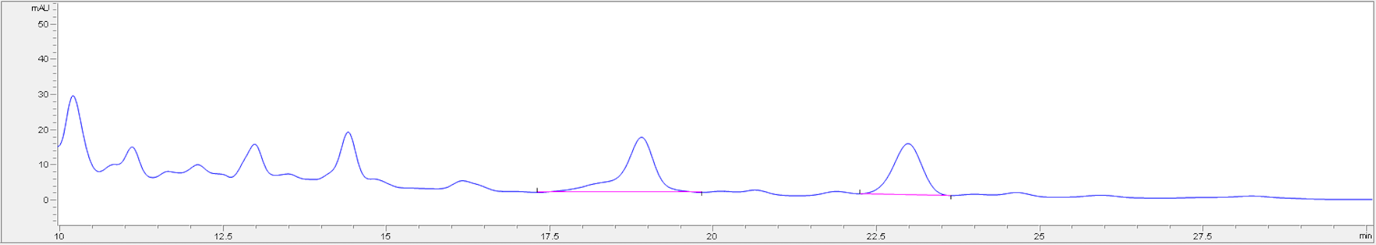


| Peak | Rt (Min) | Area (mAU*s) | Area (%) |
| --- | --- | --- | --- |
| 1 | 18.9 | 549.3 | 55.1 |
| 2 | 23.0 | 447.7 | 44.9 |

Doripenem – BlaC complex in PBS (pH 7.0) buffer


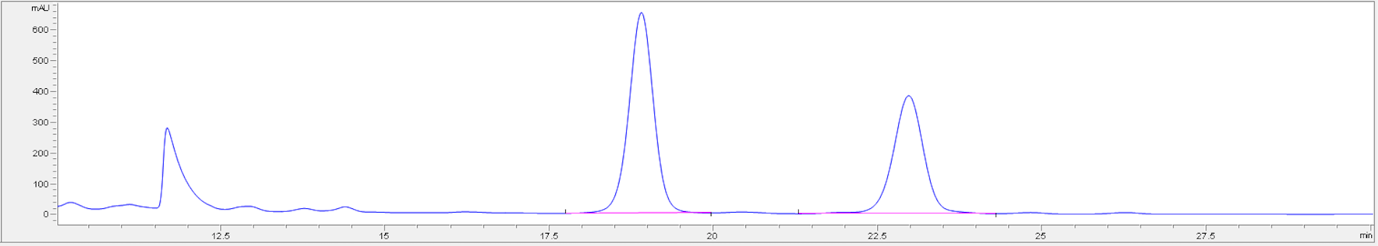


| Peak | Rt (Min) | Area (mAU*s) | Area (%) |
| --- | --- | --- | --- |
| 1 | 18.9 | 17109.7 | 58.0 |
| 2 | 23.1 | 12368.4 | 42.0 |

Meropenem under enzyme – carbapenem reaction conditions


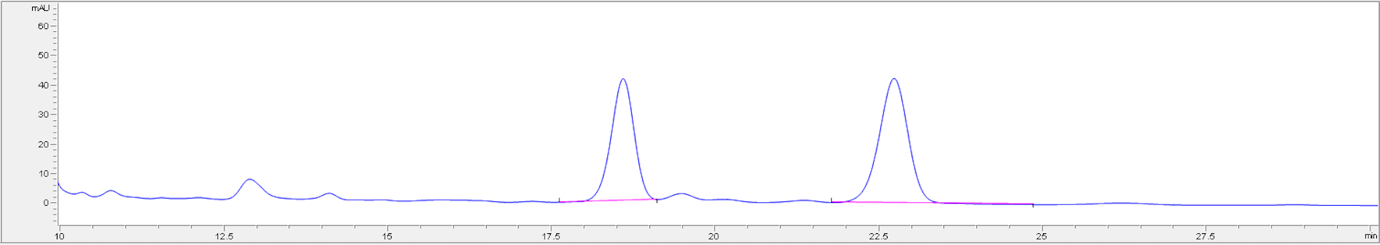


| Peak | Rt (Min) | Area (mAU*s) | Area (%) |
| --- | --- | --- | --- |
| 1 | 18.6 | 1009.3 | 43.4 |
| 2 | 22.7 | 1318.9 | 56.6 |

Doripenem under enzyme – carbapenem reaction conditions


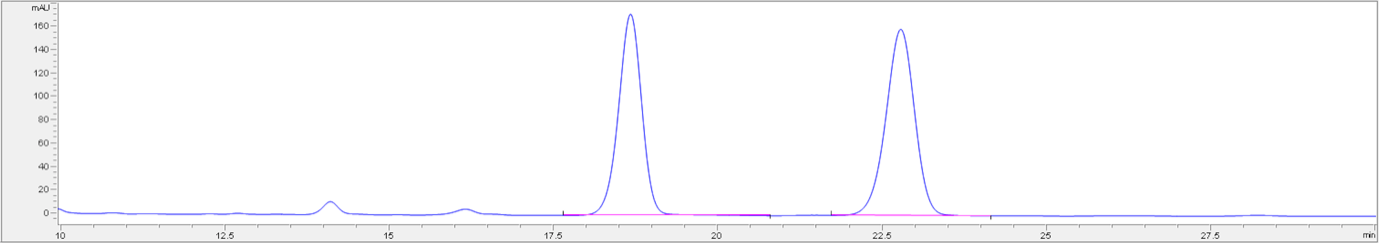


| Peak | Rt (Min) | Area (mAU*s) | Area (%) |
| --- | --- | --- | --- |
| 1 | 18.7 | 4314.2 | 46.3 |
| 2 | 22.8 | 4877.5 | 53.7 |

# DNA and amino acid sequences

DNA sequence encoding for *Mycobacterium tuberculosis* β-lactamase BlaC was taken from the literature.^4^ The gene was purchased as a double-stranded fragment (GeneArt, Invitrogen) with the following sequence. The codon of Glu166 is highlighted in red.

CTGGTGCCGCGCGGCAGCCATATGGGTGCAGATCTGGCAGATCGTTTTGCAGAACTGGAACGTCGTTATGATGCACGTCTGGGTGTTTATGTTCCGGCAACCGGCACCACCGCAGCAATTGAATATCGTGCAGATGAACGTTTTGCATTTTGCAGCACCTTTAAAGCACCGCTGGTTGCAGCCGTTCTGCATCAGAATCCGCTGACACATCTGGATAAACTGATTACCTATACCTCCGATGATATTCGTAGCATTAGTCCGGTTGCACAGCAGCATGTTCAGACCGGTATGACCATTGGTCAGCTGTGTGATGCAGCAATTCGTTATAGTGATGGCACCGCAGCCAATCTGCTGCTGGCAGATTTAGGTGGTCCTGGTGGTGGTACAGCAGCCTTTACCGGTTATCTGCGTAGCCTGGGTGATACCGTTAGCCGTCTGGATGCAGAA**GAA**CCGGAACTGAATCGTGATCCGCCTGGTGATGAACGTGATACCACCACACCGCATGCCATTGCACTGGTTCTGCAGCAGCTGGTTCTGGGTAATGCACTGCCTCCGGATAAACGTGCACTGCTGACCGATTGGATGGCACGTAATACCACCGGTGCCAAACGTATTCGTGCAGGTTTTCCGGCAGATTGGAAAGTTATTGATAAAACCGGTACGGGTGATTATGGTCGTGCAAATGATATTGCAGTTGTTTGGAGCCCGACCGGTGTTCCGTATGTTGTTGCAGTTATGAGCGATCGTGCCGGTGGTGGCTATGATGCCGAACCGCGTGAAGCACTGCTGGCGGAAGCAGCAACCTGTGTTGCCGGTGTTCTGGCATAAGGATCCGAATTCGAGCTCCGTCGAC

The gene was cloned into a pET28a vector leading to the following amino acid sequence upon expression. Glu166 is highlighted in red.

MGSSHHHHHHSSGLVPRGSHMGADLADRFAELERRYDARLGVYVPATGTTAAIEYRADERFAFCSTFKAPLVAAVLHQNPLTHLDKLITYTSDDIRSISPVAQQHVQTGMTIGQLCDAAIRYSDGTAANLLLADLGGPGGGTAAFTGYLRSLGDTVSRLDAE**E**PELNRDPPGDERDTTTPHAIALVLQQLVLGNALPPDKRALLTDWMARNTTGAKRIRAGFPADWKVIDKTGTGDYGRANDIAVVWSPTGVPYVVAVMSDRAGGGYDAEPREALLAEAATCVAGVLA*

# LC-MS analysis of BlaC and BlaC-carbapenem complexes

LC-MS chromatogram of BlaC

Mass spectrometry charged state envelope of BlaC

Deconvoluted mass spectrum of BlaC

LC-MS chromatogram of doripenem-BlaC adduct

Mass spectrometry charged state envelope of doripenem-BlaC adduct

Deconvoluted mass spectrum of doripenem-BlaC adduct

LC-MS chromatogram of Meropenem-BlaC adduct

Mass spectrometry charged state envelope of Meropenem-BlaC adduct

Deconvoluted mass spectrum of meropenem-BlaC adduct

# References

1. Wang F, Cassidy C and Sacchettini J C 2006 Crystal structure and activity studies of the Mycobacterium tuberculosis beta-lactamase reveal its critical role in resistance to beta-lactam antibiotics *Antimicrobial Agents and Chemotherapy* **50** 2762
