# Supplementary material for: Carbapenems as water soluble organocatalysts
Source: Wellcome Open Res. 2018 Aug 31;3:107. [Version 1] doi: 10.12688/wellcomeopenres.14721.1 (PMC6198258; doi:10.12688/wellcomeopenres.14721.1)
Supplement: Peer review history

# Peer review: Referee response for version 1

**DOI:** 10.21956/wellcomeopenres.16037.r34069

**Published:** 2018-10-22

**Authors:** Daniele Leonori

**Affiliations:** 1 School of Chemistry, University of Manchester, Manchester, UK

In this manuscript the authors have evaluated the use of carbapenes as asymmetric organocatalysts with the overall aim to provide effective catalytic systems for application in water. This aims stems from the known difficulty of available organocatalysts to operate under these conditions.

 Of the three carbapenems investigated, meropenem and doripenem showed promising activity for the addition of nitromethane to cinnammaldehyde. In order to stain high yields however, MeOH had to be used as the co-solvent. Under these conditions the desired product was obtained in high yield but unfortunately low enantioselectvity.

 Nevertheless, these results demonstrate a new class of organocatalysts that are able to promote organic reactions in aqueous environments which is of high relevance in the field.

I have read this submission. I believe that I have an appropriate level of expertise to confirm that it is of an acceptable scientific standard.

# Peer review: Referee response for version 1

**DOI:** 10.21956/wellcomeopenres.16037.r33795

**Published:** 2018-09-07

**Authors:** Chien-Fu Liang

**Affiliations:** 1 Department of Chemistry, National Chung Hsing University, Taichung, Taiwan

Recommendation: The manuscript is suitable to be indexed after minor revisions.

 Comments:

 In this manuscript, the authors describe the use of three water-soluble molecules as organocatalysts for Michael addition. Overall this could be a very useful transformation for carbon-carbon bond formation under environmentally friendly conditions and can readily be recycled. Additionally, they also presented several factors to illustrate the experimental results. Experiments were well designed and the results were discussed with good reasoning. The supporting data are very complete, including experimental procedure. So, it is recommended to publish this manuscript after minor revision. The following are some suggestion in this manuscript. 

1. The reaction conditions in this manuscript were performed at room temperature, I suggest the authors may try to react under the mild heating condition, and to compare the reaction time, reaction yield and enantioselectivity?
2. The substrate scope in this manuscript has only utilized the cinnamaldehyde (acyclic Michael acceptor) as a model study, I suggest the authors may put one cyclic Michael acceptor (ex: cyclohexenone) as another model study, and to compare their enantioselectivity.

I have read this submission. I believe that I have an appropriate level of expertise to confirm that it is of an acceptable scientific standard.
